# The Effect of the Addition of Tricalcium Phosphate to 5% Sodium Fluoride Varnishes on the Microhardness of Enamel of Primary Teeth

**DOI:** 10.1155/2013/486358

**Published:** 2013-05-28

**Authors:** Saeed Aedha AlAmoudi, Sharat Chandra Pani, Mohammad AlOmari

**Affiliations:** ^1^Division of Pediatric and Preventive Dentistry, Riyadh Colleges of Dentistry and Pharmacy, P.O. Box 84891, Riyadh 11681, Saudi Arabia; ^2^Division of Restorative Dentistry, Riyadh Colleges of Dentistry and Pharmacy, P.O. Box 84891, Riyadh 11681, Saudi Arabia

## Abstract

*Aim*. The aim of this study was to compare the effect of pH cycling on the microhardness of the enamel of primary human teeth treated with a conventional brown Sodium Fluoride (5% NaF) Varnish to those treated with a white Fluoride Varnish (5% NaF) enhanced with functionalized tricalcium phosphate (fTCP). *Materials and Methods*. Ninety extracted caries-free primary incisors were washed in a detergent and divided into three groups; group A received no treatment, teeth in group B were coated with Sodium Fluoride (5% NaF) Varnish, while teeth in group C were coated with 5% NaF varnish enhanced with functionalized tricalcium phosphate (fTCP). After ten days of pH cycling, the surface microhardness of the teeth was measured using a Knoop indenter. *Results*. The mean Knoop hardness number (KHN) of 5% NaF with fTCP was greater than that of 5% NaF alone while the control group had the lowest mean KHN. *Conclusion*. The results of this study suggest that the use of an additive such as fTCP to a fluoride varnish significantly improves the protective ability of the varnish on primary teeth in vitro.

## 1. Introduction

The success of topical fluoride in reducing the incidence of dental caries has been well documented [1–4]. Although professionally applied topical fluoride varnish, gel, and solutions have been shown to be effective in preventing and in arresting dental caries, varnishes are preferred by the majority of dentists because of their ease of application, reduced risk of ingestion, and patient preference [2, 4]. In recent years, researchers have tended to agree that fluoride varnishes offer an effective means of not only preventing caries, but also arresting early enamel lesions [5–7].

It has been shown that the hydrolysis of phosphates or other solids could result in the formation of calcium hydroxyapatite (HAp); however, the polymorph of tri-calcium phosphate (a-TCP or Ca_3_(PO4)_2_) is the only single solid calcium phosphate capable of expeditious hydrolysis for the formation of Hap [8]. This property is of significance as the hydrolysis and formation of HAp are accelerated in the presence of NaF ions, and the HAp formed by such hydrolysis tends to have a greater uptake of fluoride than conventional Hap [8, 9]. The functionalized form of tricalcium phosphate (fTCP) was developed as a means of improving the fluoride uptake of enamel [10, 11]. There is evidence showing that fTCP improves the fluoride uptake of permanent enamel treated with fluoridated dentifrices [11, 12]. While a recent study showed that addition of calcium and phosphate salts such as fTCP may improve the mineralization of dentin of bovine teeth [13], there is little evidence of the effect of fTCP on primary teeth.

The clinical effects of fluorides depend on the chemical compounds utilized and the methods used to apply the fluoride ion to the surface of the tooth [1]. Of the different concentrations and forms of fluoride used in varnishes, 5% NaF has emerged as one of the most popular form of fluoride varnish. Recently, a white 5% NaF varnish containing fTCP has been introduced in the market (Clinpro White, 3M corp., St. Paul, MN, USA). The use of pH cycling and enamel microhardness has been shown to be a useful in vitro tool to assess the protective effect of fluorides [14, 15]. 

 The aim of this study was to evaluate the effect of pH cycling on the microhardness of the enamel of primary teeth treated with a conventional brown 5% NaF Varnish (DuraShield, Sultan Healthcare, New York, NY, USA) with a white Fluoride Varnish enhanced with fTCP.

## 2. Materials and Methods

### 2.1. Study Design

The following is an experimental design using a microhardness tester to test the strength of the enamel after repeated cycles of demineralization and remineralization process.

### 2.2. Power of the Sample

The power of the sample was calculated using the G-Power 3.1.3 power analysis software (Universtät Kiel, Germany). The minimum required sample for the one-way ANOVA and post hoc test, with alpha of 0.05, was 20 samples in each group. Given the novelty of the fTCP varnish and lack of previous data, it was decided to use 30 samples in each group giving the group an achieved power of 0.922.

### 2.3. Preparation of the Sample

Ninety (90) extracted primary incisors free of caries were washed in a detergent following extraction. The root portions of all teeth were sealed with epoxy resin (Alteco, Quick Epoxy Adhesive, Indonesia) and then covered with red nail polish (Claire S Speed Nail Varnish, China) except for a 4 × 4 mm window area on the buccal surface. The teeth were left under running water for one minute to eliminate debris. The teeth were then randomly divided into three groups (30 teeth each). The control group (group A) comprised 30 teeth that received no protective fluoride varnish coating before subjecting them to pH cycling. Group B comprised 30 teeth coated with a brown Sodium Fluoride (5% NaF) Varnish, while group C comprised 30 teeth coated with a white fluoride (5% NaF) Varnish with functionalized tricalcium phosphate (fTCP).

### 2.4. pH Cycling and Simulation of Acid Challenge

Teeth were submitted to the formation of artificial caries by pH cycling [14] keeping the teeth in demineralizing solution (CaCl_2_ 2.2 mM, NaH_2_PO_4_ 2.2 mM, and acetic acid 0.05 M; pH of 4.5, adjusted with KOH 1 M; 15 mL per tooth) for 3 hours and in remineralizing solution (CaCl_2_ 1.5 mM, NaHPO_4_ 0.9 mM, and KCl 0.15 mM; pH of 7.0; 15 mL per tooth) for 20 hours. All the teeth were briefly washed in deionized water between solutions and placed in artificial saliva for 30 minutes at the end of the demineralization process and for 30 minutes at the end of the remineralization process (CaCl_2_ (15 mg), MgCl_2_ (5 mg), KCl (0.1 g), KSCN (10 mg), Na_2_HPO_4_ (40 mg), sodium carboxymethylcellulose (1.0 g), methylparaben (0.1 g), and water (1 L); pH of 7.0). The duration of each cycle was one day (24 hours) and the teeth were subjected to a total of 10 cycles. The demineralizing-remineralizing solutions were changed daily, and the artificial saliva was changed at every treatment.

### 2.5. Assessment of Microhardness and Evaluation of Effectiveness

**Figure 1:**
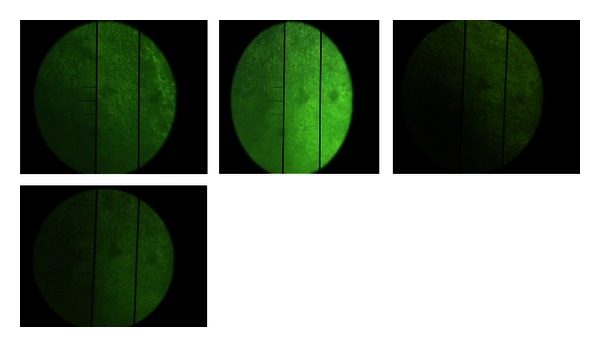
Indentation of the Knoop indenter as viewed under a microhardness tester.

The teeth were immersed in orthophthalic resin and cut along the crown's longitudinal axis through the middle of the window area to assess the hardness. The cross-sectional hardness measurements were made using a microhardness tester (MicroMet 2100 Series Microhardness Testers, USA) with a Knoop indenter and static load of 25 g and with 5 seconds of dwell time (Figure 1).

### 2.6. Statistical Analyses

The micro-hardness of the enamel of the different groups after pH cycling was compared using the one-way ANOVA, and the Scheffes post hoc test was used to determine the significance of intergroup variation.

## 3. Results

The mean and standard deviations and error of means of the microhardness of different groups after pH cycling are shown in Table 1. The mean Knoop hardness number (KHN) of group C (5% NaF with fTCP) was greater than that of group B (5% NaF) while the control group (group A) had the lowest mean KHN. The one-way ANOVA found these differences to be significant (*P* < 0.05). 

The Scheffes post hoc test, used with a difference set at *P* < 0.05 to determine intergroup variation, showed that group C (5% NaF with fTCP) had significantly greater microhardness than the control group (Table 2). However, group B (5% NaF) showed microhardness that was intermediate to group C (5% NaF with fTCP) and the control group (Table 2).

## 4. Discussion

The efficacy of topical fluorides in general and fluoride varnishes in particular in reducing dental caries has been extensively documented [2, 16–18]. Since the advent of the first varnishes, researchers have strived to improve both efficacy and delivery of fluorides in varnishes [3, 10, 13]. Since the functional form of tricalcium phosphate (fTCP) has been shown to improve the uptake of fluoride and aid in remineralization of carious lesions in both enamel and dentin when incorporated into fluoride dentifrices [10–12], this study aimed to evaluate if a similar protective effect existed when fTCP was incorporated into a fluoride varnish. 

The results of this study indicate that there is highest microhardness in the surface of the enamel group treated with NaF with fTCP. Surface microhardness (SMH) has been used as a reliable indicator of the efficacy of fluorides and is an effective measure of the overall impact of the mineralization on the tooth [11, 14, 15]. Although SMH has often been used in addition to tests such as demineralization depth [19, 20] or loss of fluorescent lesion area [21], it has been used alone in experiments to evaluate the overall protective effect of different fluoride regimens [14, 22]. While our study showed a significantly higher SMH for the varnish with fTCP, it also suggested that NaF varnishes with and without fTCP had significantly higher protective effect than an unprotected tooth.

A recent study showed that initial carious lesions treated with fluorides that had fTCP added to them had a greater remineralization than conventional 5% sodium fluoride. This seems to suggest that addition of calcium and phosphate salts such as fTCP may improve the mineralization of dentin of bovine teeth [13].

Much of the research on fTCP has been done using fluoride dentifrices. Karlinsey et al. used a hybrid material comprised of beta-tri-calcium phosphate (beta-TCP) and sodium lauryl sulfate (SLS) prepared using a mechanochemical process, examined using particle size analysis, found that microhardness values increased up to 30% greater than fluoride alone [10]. 

The addition of fTCP has not been the first attempt to incorporate calcium into fluorides. Initial research showed that the provision of dissolved fluoride was the key to successful therapy. The source of this fluoride could either be fluorapatite or calcium fluoride- (CaF_2_-) like precipitates, which are formed on the enamel and in the plaque after application of topical fluoride [23]. However, results with varnishes incorporating calcium fluoride have not been promising [24]. In their comparison of CaF modified varnish with 5% NaF, Ferreira et al. found that both varnish formulations tested produced similar clinical effects [24].

In spite of several studies indicating the benefits of adding fTCP [10, 12, 13], there have been those who have argued that casein phosphopeptide-amorphous calcium phosphate (CPP-ACP) may prove to be a more effective alternative to fTCP [25]. However, there have been arguments that both CPP-ACP paste and tricalcium phosphate increase the hardness of the teeth in vitro when added to products containing fluoride [26].

While the mechanism of action of fluoride is based on the interaction with calcium hydroxyapatite, studies have shown that the structure of enamel could influence the ultimate clinical efficacy of the fluoride [1]. Studies that have yielded positive results on in vitro bovine or rat models have often failed to produce similar results in human mouths [27, 28]. In this respect, there is a need to assess the impact of fluoride on primary enamel. Gatti et al. (2011) suggested that while the overall effects of fluoridated dentifrices on primary teeth were similar to those on permanent teeth, there were certain differences in the primary teeth when the teeth were tested in vitro [14]. Primary enamel has a far greater amount of structure-less enamel making the transfer of fluoride ions across the crystals less pronounced than in permanent teeth. In this respect, the formation of new hydroxyapatite from the hydrolysis and uptake of tricalcium phosphate salts may be significant [8]. Our results, which show a significantly higher surface microhardness of teeth that were exposed to 5% NaF with fTCP, could be a manifestation of new hydroxyapatite formation.

The results of the current study are on an in vitro model, and while they reflect favorably on the effect of the addition of fTCP to 5% NaF, the results need to be validated clinically. Products such as Calcium Fluoride, which were theoretically labeled as effective in in vitro research have proven to be no more effective than regular 5% NaF in clinical studies [25]. Therefore, there is a need for greater clinical studies and a need to compare the effect of fTCP to that of other products such as CPP-ACP, and these could serve as the basis for future research.

## 5. Conclusion

The results of this study suggest that the use of an additive such as fTCP to a fluoride varnish significantly improves the protective ability of the varnish on primary teeth in vitro. However, the results of this study are based on surface microhardness readings, and would need to be clinically validated.

## Figures and Tables

**Table 1:** Surface microhardness (SMH) values of different groups*.

	Mean SMH	Std. deviation	Std. error	Minimum	Maximum	*F*	Sig**
Control	1862.23	646.717	118.073	1278.00	3818.00		
fTCP	2380.46	709.596	129.554	1543.00	4117.00	4.848	0.010***
5% NaF	2185.33	591.580	108.007	1416.00	3967.00		
Total	2142.67	678.653	71.536	1278.00	4117.00		

*SMH values measured in KHN.

**Significance calculated using the one-way ANOVA.

***Differences significant at *P* < 0.05.

**Table 2:** Post hoc test indicating the significance of difference in SMH values of different groups*.

Group	*N*	Subset for alpha = 0.05	
		1	2
Control	30	1862.23	
5% NaF	30	2185.33	2185.33
fTCP	30		2380.46
Sig.		0.164	0.512

Means for groups in homogeneous subsets are displayed.

Used harmonic mean Sample size = 30.000.

*Calculated using the Scheffe post hoc test.
